# Willingness to pay for sheep traits and their heterogeneous effects on prices: Evidence from primary livestock markets in Ethiopia

**DOI:** 10.1371/journal.pone.0308651

**Published:** 2024-09-19

**Authors:** Asresu Yitayew, Girma T. Kassie, Awudu Abduali, Zerihun Nigussie

**Affiliations:** 1 Amhara Region Agricultural Research Institute, Bair Dar, Ethiopia; 2 International Centre for Agricultural Research in the Dry Areas (ICARDA), Rabat, Morocco; 3 Department of Food Economics and Food Policy, University of Kiel, Kiel, Germany; 4 College of Agriculture and Environmental Sciences, Bahir Dar University, Bahir Dar, Ethiopia; JNKVV: Jawaharlal Nehru Krishi Vishwa Vidyalaya, INDIA

## Abstract

We test the hypothesis whether levels of key traits of sheep heterogeneously affect market prices of sheep in a rural setting. Feasible generalized least squares and (un)conditional quantile regression estimations were made on a dataset of 1153 sheep transactions in two primary small ruminant markets in the Amhara region of Ethiopia. The empirical results show that animal traits affect the observed prices of sheep differently, but only partly explain the sheep price differences. Our results also reveal that in addition to animal traits, the type of buyers and seasonality of sheep marketing cause heterogeneity in the observed prices. These findings imply that targeting the animal traits demanded by the market and access to price information that enables farmers to respond to the seasonal changes in livestock markets are essential to increase the income of sheep keepers.

## 1. Introduction

Evidence based market development interventions can improve livelihoods of millions of poor rural households in Ethiopia. Sheep production is a vital component of agriculture in rural Ethiopia where mixed crop-livestock production is the mainstay of livelihood serving as a source of subsistence and cash income for smallholder livestock keepers [1, 2]. However, livestock production in Ethiopia is generally constrained by different challenges that undermine the production and marketing performance of livestock keepers [3]. Livestock markets are typically characterized by asymmetric information and short market durations that limit the bargaining power of farmers. For example, farmers’ bargaining power decreases when they are forced to rush to transact due to poor market infrastructure [4]. Similarly, the lack of access to reference price information decreases their market performance [5]. Therefore, livestock keepers do not usually benefit from the improvements made in the agricultural markets and marketing [4].

Most of the sheep production happens in rural parts of the country, and yet little is known about what determines the price of sheep in Ethiopian livestock markets. The little documented effort that analyzed the pricing of sheep focused on the spatial and temporal aspects of the markets and characteristics of buyers and sellers with little or no regard to traits of the sheep being marketed [6, 7]. We could not, in fact, find any study that analyzed the heterogeneity of the effects of sheep traits on the price buyers pay for the animals. The lack of this information has created a disconnect between what buyers focus on when purchasing sheep and what agricultural extension efforts focus on in trying to improve the participation of sheep keepers in the market [8, 9]. Hence, given the importance of sheep in the livelihoods of rural communities in Ethiopia, it is imperative to understand the way price is determined for different types of sheep by different types of buyers. Such studies will inform policy and strategy formulation that aim at improving the livelihoods of sheep keepers in the rural parts of the country through a more informed market development initiative.

Previous studies have shown that livestock markets in Ethiopia are not competitive, and hence the observed price of the animals cannot be fully explained by characteristics of the animals being transacted only [1, 10]. Sheep prices are a function of the sheep traits as well as the characteristics of the buyers, market, and the season. A buyer evaluates each of the traits of the sheep, attaches implicit prices to them, and decides how much to pay for the sheep after a long and intricate valuation process [2].

Given that smallholder farmers are less likely to produce sheep primarily for selling in a market-oriented manner, their market supply decisions are governed by cash need, feed availability, and sociocultural factors [1, 2, 10]. It is important to note that because the purpose of market participation decisions by buyers is quite different, they are less likely to have similar preferences for animal traits. The difference in preferences for animal traits drives the heterogeneity in buyers’ willingness to pay for the traits. The studies on small ruminant market behavior have therefore concluded that animal attributes, types of buyers, marketplaces, and seasons of transaction are important factors that influence livestock price formation [1, 10–12].

In a competitive market setting, Lancaster’s theory of value posits that the value of a quality differentiated product emanates from its characteristics [13]. Accordingly, the willingness to pay for a certain sheep trait, for example, is considered a hedonic price, so that the total price of the sheep can be broken down into a fixed price element and the values of its traits [14]. The hedonic pricing method we employ in this study is becoming the standard approach in estimating the implicit values buyers attach to animal traits. The implicit values of the traits are quantified through the observation of the consumption behavior of buyers in actual or hypothetical markets. Implicit prices measure willingness to pay for the sheep traits and predominantly depend on consumers’ perceived utility from the traits [13–15].

Previous studies that analyzed small ruminant market prices estimated hedonic models in similar settings with the assumption that the trait levels and other factors affect the distribution of observed prices in a homogenous pattern [1, 2]. Such assumptions, however, exclude the heterogeneous effects of the traits across the distribution of observed prices. Estimating the hedonic price model in livestock pricing analysis through ordinary least squares at the conditional mean, without accounting for differences in buyers’ preferences for animal attributes, is, therefore, more likely to mask important policy implications. This is because the model assumes that the higher-valued animal traits behave like the lower-valued traits possibly hiding the potential heterogeneity in prices. Revising the assumptions behind the models and, hence, reformulating the models to estimate the observed prices over traits and other key factors will be essential not only for reliable characterization of price formation in such non-competitive markets but also for effective targeting of market development interventions.

The primary objective of this study is, therefore, to examine the heterogeneous effects of sheep traits on market prices of sheep in two rural markets in the Amhara region, using quantile regression models [16]. The study makes an important empirical contribution to the relevant body of knowledge. We depart from the common practice of assuming homogeneous effects of traits and other factors on the continuum of price and investigate the heterogeneous effects. In line with our hypotheses, we find that the levels of sheep trait affect price differently, and the willingness to pay for sheep by various types of buyers is heterogeneous. The deriving factors of heterogeneity in the observed prices are sheep traits, different types of buyers, and time of transactions. For example, buyers are willing to pay higher prices per kilogram for sheep with ‘good’ body condition than for one with ‘very good’ body condition in all price ranges. Buyers pay the highest per kilogram for sheep marketed at the age of less than half a year (cf. less than six months), and they tend to pay the lowest for those aged above three years (cf. less than six months).

The remainder of the paper is organized as follows. The next section presents the analytical framework and specification of the econometric model we employed. Section 3 describes the market context and the data used in the analysis. Section 4 presents the empirical results and discussion, while the last section presents the lessons drawn from the empirical findings of the study.

## 2. Analytical framework

Buyers’ perceived utility from the traits of the sheep they purchase is revealed through the price they pay for the sheep. To relate this perceived utility with the revealed preferences, we estimate a hedonic price model following [11, 14] in a way that accounts for the imperfect competitiveness of livestock markets. Economic theory hardly informs about the functional form of hedonic price model and yet places few restrictions [17, 18]. In line with previous empirical studies and the dependence of observed prices on the traits of the sheep, we begin with the basic log-linear parametric specification [1] given as:

ln(Y)=α+βX+ε [1]

where In(*Y*) is the natural log of the observed price of sheep; ***X*** includes traits of the sheep, types of buyers, and periods of transactions; *β* denotes the parameters to be estimated–including the implicit price of sheep traits; ε denotes independent and identically distributed error term with zero mean and constant variance. We estimate the hedonic model using OLS and two heteroscedastic consistent estimators.

Analysis of the determinants of heteroscedastic prices can be done with OLS estimators and it usually results in good parameter estimates. The problem that arises from using OLS in a heteroscedastic model is that the estimated variance-covariance matrix is not consistent. One way of addressing this problem is using weighted least squares (WLS) method where parameter estimates are obtained by minimizing a weighted sum of squares of residuals where the weights are inversely proportional to the variance of the errors. Another method is the feasible generalized least square (FGLS) which is a two-stages estimator where the first stage estimates the variance structure of the errors, and the second stage estimates the coefficients of the model accounting for the variance structure of the error terms. WLS and FGLS are expected to generate more efficient estimates than OLS for large samples. And FGLS is expected to generate more efficient estimates than WLS [19]. We estimated linear regression models using all three methods to show the gradual progress in quality of the estimates. The FGLS resulted in the most efficient estimates and hence we argue that our approach was justified as the data were heteroscedastic.

We begin with the general specification of heteroscedasticity given as $Var\left(\varepsilon_{i}\right)=\sigma_{i}^{2}=\sigma^{2}\psi_{i},$ such that

$$\text{E}(\varepsilon \varepsilon^{\prime })=\sigma^{2}\left[\begin{array}{ccc} \psi_{1} & 0 & 0\\ 0 & \psi_{2} & 0\\ 0 & 0 & \psi_{3} \end{array}\right]=\sigma^{2}\Omega$$

$$\sigma^{2}\Omega^{-1}=\left[\begin{array}{ccc} \psi_{1}^{-1} & 0 & 0\\ 0 & \psi_{2}^{-1} & 0\\ 0 & 0 & \psi_{3}^{-1} \end{array}\right]$$

Given that $\Omega^{-1}=P^{\prime }P$ *P* is n x n matrix whose 1- diagonal element is $1/\sqrt{\psi_{i}}$.

Then, by pre-multiplying *Y* and *X* with P we find

$$Y^{*}=\text{P}*Y\left[\begin{array}{l} Y_{1}/\sqrt{\psi_{1}}\\ Y_{2}/\sqrt{\psi_{2}}\\ Y_{n}/\sqrt{\psi_{n}} \end{array}\right]$$

and

$$X^{*}=\text{P}*X\left[\begin{array}{c} 1/\sqrt{\psi_{1}}\;\;\;x_{11}/\sqrt{\psi_{1}}\;\;\;\cdots \;\;\;x_{1k}/\sqrt{\psi_{1}}\\ 1/\sqrt{\psi_{2}}\;\;\;x_{21}/\sqrt{\psi_{2}}\;\;\;\cdots \;\;\;x_{1k}/\sqrt{\psi_{2}}\\ 1/\sqrt{\psi_{n}}\;\;\;x_{n1}/\sqrt{\psi_{n}}\;\;\;\cdots \;\;\;x_{nk}/\sqrt{\psi_{n}} \end{array}\right]$$

Following this, we re-specify Eq [1] as an WLS estimator as follows:

$$\text{P}*[\text{ln}(Y)]=P*\beta X+P*\varepsilon \Rightarrow \text{ln}\left(Y^{*}\right)=X^{*}\beta +\varepsilon^{*}$$ [2]

We also hypothesize that the trait levels have different effects along the distribution of the price of sheep. Therefore, instead of estimating the hedonic price model only at the conditional mean *E*(In (Y)|X)as in OLS, we use quantile regression (QR). QR model estimates the relationship between the price of the sheep [In(*Y*)] and the traits and other regressors (*X*) at different quantiles (*q*) of the price distribution. The quantile, *q*, is defined as the split of the price of sheep *Y* into the proportion *q* below and 1-*q* above the estimated lines.

There are different formulations of quantile regression. These include conditional quantile regression (CQR) [20] and unconditional quantile regression (UQR) [21]. The conditional QR shows the effect of a given independent variable on the price sheep given specific values of the other explanatory variables. Hence, the coefficients show the relative importance of the trait considered for a specific group of sheep characterized by the average values of the independent variables. The unconditional QR estimates the importance of the trait or other explanatory variables considered in explaining sheep price for the entire sample.

As discussed by [20], the *q*_τ_ quantile is given by $q=F_{Y}\left(\mu_{q}\right)$ and $\mu_{q}=F_{Y}^{-1}(q)$ where $F_{Y}^{-1}(q)$ is the inverse of the cumulative distribution function of *Y*. Quantile *q* can be defined as a linear regression $Q_{Y}=(q|X)=X\beta_{q},\beta_{q}=\beta +v(.)F_{Y}^{-1}(q),$ and where the (*q*) indicates that the parameters are for a specified quantile *q* and vary with *q* due to effects of the τ^*th*^ quantile of the distribution. The τ^*th*^ quantile estimator can be specified in such a way that both under-prediction $\left(Y>X^{\prime }\beta \right)$ and over-prediction $\left(Y<X^{\prime }\beta \right)$ are accounted for as follows:

$$Q\left(\beta_{q}\right)=\sum_{i:Y\geq {X^{\prime }}^{\beta }}^{N}q\left|Y-X^{\prime }\beta_{q}\right|+\sum_{i:Y<X^{\prime }\beta }^{N}(1-q)\left|Y-X^{\prime }\beta_{q}\right|$$ [3]

Unlike the CQR, the UQR consists of the recentered influence function (RIF) for analyzing the unconditional partial effects of the explanatory variables on different quantiles of the outcome variable. We define the RIF of the τ^*th*^ quantile distribution of the price of sheep *Y* as follows:

$$RIF\left(Y;q_{\tau },F_{Y}\right)=q_{\tau }+\frac{\tau -1\left\{Y\leq q_{\tau }\right\}}{f_{Y}\left(q_{\tau }\right)}.$$ [4]

where *q*_τ_ is the τ^th^ quantile distribution of the price of sheep; *F*_*Y*_ is the cumulative distribution function of *Y*; $1\left\{Y\leq q_{\tau }\right\}$ is an indicator function taking the value one when its argument is correct or zero otherwise, *F*_*Y*_(*q*_τ_) is the density of *Y* evaluated at *q*_τ_. By transforming the nonlinear Eq [4], we re-specify the hedonic pricing model in Eq [1] as a linear RIF regression as follows:

$$E\left[RIF\left(Y;q_{\tau }\right)|X\right]=X^{\prime }\beta +\epsilon$$ [5]

where the notations are the same as Eq [1]; *β* denotes the marginal effects of animal and buyer attributes and periods of transactions in quantile *q*_*τ*_. As shown in Eq [5], by using RIF as the dependent variable, we use OLS to estimate unconditional marginal effects on quantiles of price of sheep. It is also important to be cautious about the probability density and the standard errors of unconditional quantile regression [22]. Following recent studies that applied unconditional quantile regression models [23], we estimated the probability density using the Gaussian Kernel and the Silberman optional bandwidth, while we computed the standard errors using the bootstrapping method.

## 3. Data and market context

Data used for this study were collected in two primary livestock markets in the Amhara region of Ethiopia in 2010. We collected data on 1153 sheep transactions at *Gonji* (457 transactions) and *Quarit* (696 transactions) livestock markets. These markets are local and primary markets located 20 and 40 kilometers away from Adet market (secondary market), and 60 and 80 kilometers away from Bahir Dar market (tertiary market), respectively. The data collected included sheep traits (sex, age, live weight, body condition, and coat color), selling price, types of buyers, and period of transactions. Trained enumerators measured the live weight of the animal using an instrument that scales the dressing percentage of animals, and visually scored body condition following standard guidelines [24].

Based on their main purpose of purchasing the sheep, we classified the sheep buyers into three groups, i.e., producers, consumers, and traders. Producers buy sheep mainly for breeding and to a limited extent for reselling purposes, while traders, including collectors, purchase solely for reselling (profit-making). Traders are those who buy and sell to take advantage of price differences over time and across markets. They usually buy sheep in primary markets and sell them in secondary and tertiary markets for higher prices. In some cases, traders buy sheep and then sell them in the same market.

The markets surveyed lack basic infrastructure, such as sheds, water troughs, feed lots, and information displays. The markets set only two days a week for not more than a couple of hours. As they have access to alternative information communication technologies, traders are usually more informed than producers [farmers], indicating the presence of information asymmetry in rural livestock markets [4]. Information asymmetry can result in higher price differences between markets [25]. In addition, short market durations and lack of sheds [that further reduce farmer’s duration in the market] limit farmers’ options and, hence, undermine their bargaining power.

## 4. Results and discussion

### 4.1. Descriptive statistics

Table 1 presents descriptive statistics of the price and traits of sheep marketed. The average price of the sheep that weigh on average 18 kilograms is 156 Ethiopian Birr (Birr). Birr is the official Ethiopian currency, and the average exchange rate at the time of the survey was 1 USD to 11.619 Birr. Most of the sheep (78%) supplied to the markets are too young, aged a year or less. In addition, female sheep with mixed coats dominate the sheep population supplied to the markets. The coat colors observed are white, brown, and mixed, the last two being quite dominant. Table 1 also presents the types of the buyer and the periods of transaction. Most of the market participants (47%) are consumers, followed by producers (35%) and traders (18%). The frequency of sheep transactions is highest during festive periods, specifically Ethiopian Easter usually celebrated in April, while the smallest number of sheep transactions is during the non-festive periods, particularly in October and February.

**Table 1:** Descriptive statistics of the observed sheep transactions.

Variable	Mean	St. Dev.
***Continuous variables***		
Price (Birr/head)	156.12	75.40
Live weight (kg)	17.91	7.79
***Categorical variables***	Col %	
**Sex of sheep**		
Female sheep	58.89	
Male sheep	41.11	
**Age of sheep**		
Less than six months	58.11	
B/n half and one year	19.69	
B/n one and two years	9.11	
B/n two and three years	4.94	
Above three years	8.15	
**Body condition**		
Good	47.27	
Very good	52.73	
**Coat color of sheep**		
Mixed	58.28	
White	12.32	
Brown	29.40	
**Buyers**		
Producer	34.9	
Consumer	46.87	
Trader	18.22	
**Months of observation**		
September	10.55	
October	6.36	
November	4.27	
December	6.54	
January	6.89	
February	3.31	
March	11.42	
April	17.35	
May	9.85	
June	7.15	
July	6.45	
August	9.85	

Notes: Female sheep, less than six months, good body condition, mixed coat color, producer, and September transaction are benchmark categories for comparison in the estimation.

Summary of the traits of sheep for each type of buyer is presented in Table 2 below. Looking into the associations between the different traits and the prices paid by the buyers reveals that consumers [those who purchase for consumption purposes] pay higher prices for all types of sheep compared to traders and producers. On the contrary, traders [those who purchase for reselling purposes] pay considerably less to all types of animals compared to consumers and traders.

**Table 2:** Summary of price paid for different types of sheep by buyers.

Variable	Producer	Trader	Consumer
Very thin sheep	92.84	93.63	138.44
	(23.95)	(35.54)	(83.68)
Thin sheep	132.38	122.57	146.41
	(44.03)	(31.99)	(58.99)
Moderate sheep	216.79	202.74	227.47
	(57.99)	(55.11)	(81.39)
Fat sheep	249.40	284.46	340.14
	(40.26)	(82.68)	(90.13)
Female sheep	168.79	147.21	204.01
	(68.79)	(70.24)	(102.53)
Male sheep	133.02	131.49	182.34
	(62.86)	(48.79)	(100.09)
Less than six months	118.30	119.58	147.51
	(40.92)	(37.08)	(66.26)
B/n half and one year	163.59	143.52	165.60
	(65.17)	(47.68)	(60.76)
B/n one and two years	201.45	200	257.12
	(45.65)	(86.43)	(110.85)
B/n two and three years	232.89	245.5	334.29
	(45.16)	(87.96)	(123.52)
Above three years	256.52	260	300.19
	(55.46)	(67.12)	(92.35)
Good	133.99	134.86	127.55
	(59.16)	(56.95)	(49.07)
Very good	167.27	149.59	220.09
	(70.05)	(68.22)	(105.17)
Mixed	156.72	142.33	198.93
	(67.63)	(66.08)	(108.32)
White	162.94	141.09	171.5
	(63.02)	(68.40)	(79.36)
Brown	163.74	133.49	194.34
	(75.35)	(47.09)	(94.11)

*Note*: Figures in parentheses are standard deviations. The number of sheep sold to different market actors is shown in Table A1 in the S1 Appendix.

### 4.2. Determinants of sheep prices

Before estimating the hedonic price model, we checked the distribution of the error terms using the Breusch-Pagan test and found that they were heteroscedastic (see Table A2 in the S1 Appendix). Table 3 presents the relative importance of the factors that affect the price of sheep based on model results using specification [2]. We report the estimates of ordinary least squares (OLS) in column [1], weighted least squares (WLS) in column [2], and feasible generalized least squares (FGLS) in column [3]. As expected, FGLS has resulted in most efficient parameter estimates as observed through the magnitude of the standard errors. Therefore, the discussion in this section is based on the FGLS estimator.

**Table 3:** Estimates of hedonic price model using OLS, WLS and FGLS estimators.

Variable	OLS	WLS	FGLS
	(1)	(2)	(3)
**Dependent variable = ln of price**			
Live weight	0.067^***^	0.067^***^	0.067^***^
	(0.005)	(0.008)	(0.004)
Live weight squared	-0.001^***^	-0.001^***^	-0.001^***^
	(<0.001)	(<0.001)	(<0.001)
Male sheep	0.032^*^	0.020	0.020
	(0.017)	(0.028)	(0.016)
B/n half and one year	-0.091^***^	-0.134^***^	-0.134^***^
	(0.019)	(0.033)	(0.018)
B/n one and two years	0.020	0.056	0.056^***^
	(0.021)	(0.038)	(0.018)
B/n two and three years	0.110^***^	0.136^***^	0.136^***^
	(0.028)	(0.040)	(0.025)
Above three years	0.089^***^	0.128^***^	0.128^***^
	(0.025)	(0.048)	(0.024)
Very good	0.197^***^	0.198^***^	0.198^***^
	(0.021)	(0.042)	(0.021)
White	0.021	0.029	0.029^**^
	(0.016)	(0.018)	(0.014)
Brown	-0.031^***^	-0.047^***^	-0.047^***^
	(0.012)	(0.015)	(0.011)
Trader	-0.029^**^	-0.029	-0.029^**^
	(0.013)	(0.018)	(0.013)
Consumer	0.057^***^	0.076^***^	0.076^***^
	(0.014)	(0.022)	(0.017)
**Month of purchase**			
October	0.012	0.024	0.024
	(0.027)	(0.024)	(0.026)
November	0.009	0.009	0.009
	(0.033)	(0.038)	(0.040)
December	-0.053^**^	-0.046	-0.046^*^
	(0.027)	(0.033)	(0.026)
January	-0.072^***^	-0.049^**^	-0.049^**^
	(0.026)	(0.024)	(0.024)
February	-0.039	-0.026	-0.026
	(0.037)	(0.039)	(0.040)
March	0.046^**^	0.019	0.019
	(0.021)	(0.05)	(0.023)
April	0.057^***^	0.053	0.053^**^
	(0.019)	(0.037)	(0.021)
May	-0.009	-0.020	-0.020
	(0.022)	(0.025)	(0.016)
June	0.040	0.057	0.057^**^
	(0.026)	(0.035)	(0.025)
July	-0.003	-0.011	-0.011
	(0.027)	(0.029)	(0.033)
August	0.029	0.007	0.007
	(.023)	(0.021)	(0.022)
Market fixed effect	-0.091^***^	-0.091^**^	-0.091^***^
	(0.024)	(0.042)	(0.023)
Constant	4.08^***^	4.16^***^	4.16^***^
	(0.061)	(0.114)	(0.052)
R-squared	0.686	0.755	0.755
Observations	1153	1153	1153
aic	10.66	-84.80	-84.80
bic	136.91	41.45	41.45

Notes: We included market fixed effects to control for unobserved market specific attributes in all estimations. Figures in parentheses are standard errors; and *, **, and *** denote statistical significance at the level of 5%, 1%, and 0.1% statistical error, respectively.

The results reveal that live weight and body condition positively and significantly affect the observed price of sheep. This implies that traits related to the volume and quality of meat are important in determining the price of sheep. These findings are comparable to those reported by other revealed preference-based livestock pricing studies [11, 26]. However, the quadratic term of weight has a negative and statistically significant effect on the price of sheep. The quadratic effect implies that animal weight positively impacts prices up to 33.5 kilograms and then starts to have a negative effect on price, which is consistent with earlier studies, see for example [10, 26].

The results also show that animal traits, such as age and coat color, affect the observed prices of sheep, while the sex of the sheep does not have a significant relationship with price. The effect of age and color of the sheep on the observed price of the sheep is mixed. Compared to sheep marketed at the age of less than six months, older sheep fetch higher prices except those aged between 6 months and 1 year, which command a significantly lower price. Specifically, sheep aged between two and three years (cf. less than six months) command the highest price. Compared to mixed-coat sheep, white- and brown-coated sheep are sold at a premium and discount, respectively. These findings differ from results reported on sheep markets that are connected to large cities, such as the capital Addis Ababa in Ethiopia [26] and goat marketing in Benin [27].

The results also reveal that the willingness to pay for sheep varies between different types of buyers. In line with our observation above, consumers pay higher prices, while traders pay significantly less per sheep compared to producers. Marketing seasons in which transactions take place also play a significant role in sheep pricing, as is the case in other agricultural markets in Ethiopia [28]. Sheep command high price during Ethiopian Easter (April) and Apostles’ Fast (June). The price of sheep declines from December to January. This happens because the quantity of mutton demand decreases during the Nativity Fast that is widely observed before Ethiopian Christmas and Epiphany.

### 4.3. Conditional marginal effect of animal traits

**Fig 1:**
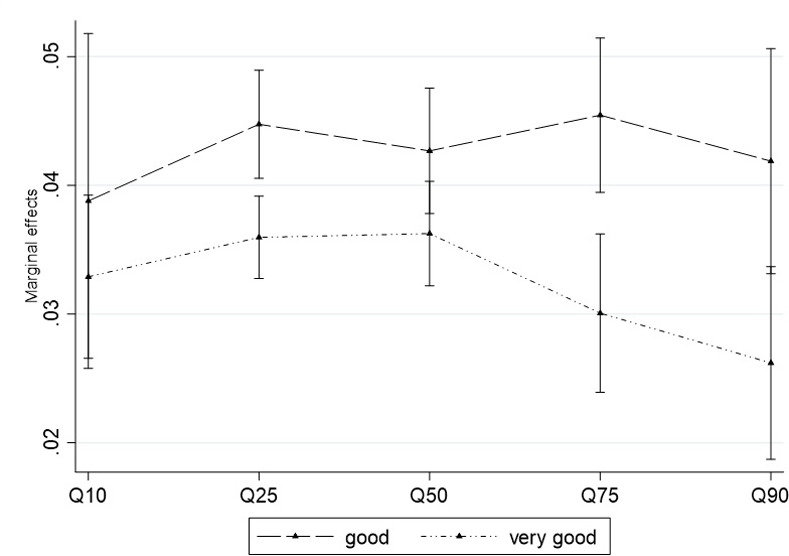
The marginal effects of live weight on sheep price conditional on body condition.

**Fig 2:**
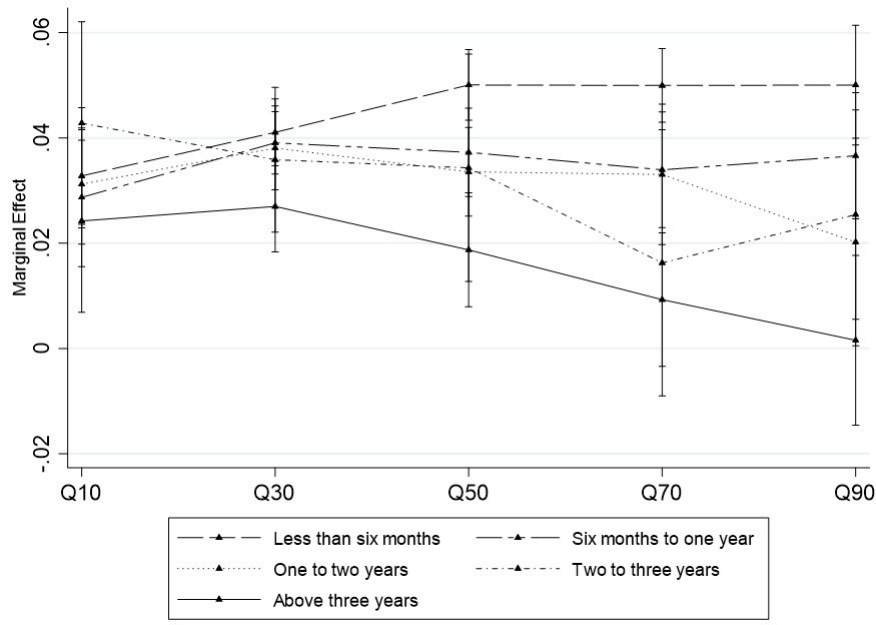
The marginal effects of live weight on price of sheep conditional on age of the sheep.

In this section, we present the marginal effects of animal traits on price of sheep estimated using CQR (equation 3). Conditional quantile partial effects of changes in the traits of sheep on distribution of sheep price at a specific value given other factors that influence pricing. The conditional partial effects of the sheep traits can inform designing and implementation of improved husbandry practices and marketing. Hence, we estimate the marginal effects of live weight on the observed price of sheep, conditional on age and body condition of the sheep. The results are shown in Figs 1 and 2.

Fig 1 presents the marginal effects of live weight on price, conditional on body condition of the sheep. The marginal effects of live weight on price are much more pronounced when the sheep are in *good* body condition than when they are in *very good* body condition. Consistent with the quadratic effects of live weight discussed above, animals weighing above 33.5 kilograms and in very good body condition are more likely to get fatty. This finding therefore implies that buyers are willing to pay a higher price per kilogram for sheep which are not fatty (cf. fatty ones) over all price ranges. This is possibly because consumers prefer red meat over fat.

Fig 2 shows the marginal effects of live weight on sheep price, given the age category of the sheep. The figure reveals that the marginal effect of live weight on sheep price is quite different across the different sheep age groups. Buyers pay the highest per kilogram for sheep marketed at the age of less than half a year, followed by sheep marketed at the age of between half a year and one year. In contrast, they tend to pay the lowest per kilogram for sheep marketed at the age of more than three years. This finding implies that improving the percentage of dressing for young sheep using improved fattening technologies could improve the price sheep keepers receive.

### 4.4. Heterogeneity effects of animal and buyer attributes on prices

**Fig 3:**
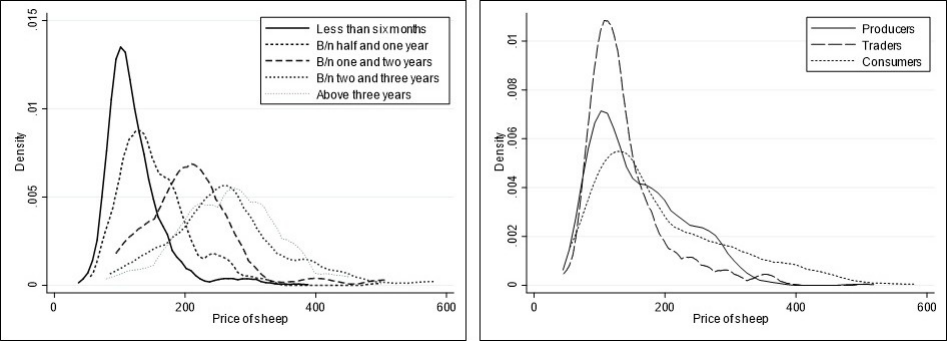
Distribution of sheep prices for various age categories (left) and different types of buyers (right).

In addition to understanding the relationship between sheep and buyer attributes and the observed price, policy makers and practitioners are interested in information on heterogeneous effects of attributes on prices. Fig 3 shows the distribution of the prices of sheep for different age categories and buyers. Fig A1 in the S1 Appendix also shows how the influence of each animal trait varies over quantiles and how this influence changes considerably across quantiles based on the OLS estimations. The price distribution shows less variation for young sheep compared to older sheep. Generally, however, as age increases, the price of sheep also increases. The kernel densities for the different buyers exhibit skewness to the right with comparable variance. It is, however, clear that the average price paid by consumers is higher ‐ in line with the discussions above. These results indicate that the heterogeneity in the prices paid for the sheep in the markets emanates from the different levels of preferences for the traits across the price continuum.

Table 4 presents heterogeneous effects of sheep traits and buyer types on the distribution of sheep price using the UQR model with market fixed effects. The results show that the marginal effects of live weight are statistically significant in all price quantiles [ranging from 10^th^ to 90^th^]. The marginal effects are all positive except in the 90^th^ price quantile. This reveals preference heterogeneity such that at the lower and middle quantiles, higher live weight is associated with higher price and heavier sheep are priced lower at the upper end of the price distribution.

**Table 4:** Estimates of hedonic price model using UQR.

	Quantiles				
Variable	10th	30th	50th	70th	90th
**Dependent variable (ln of price)**					
Live weight	0.079^***^	0.116^***^	0.104^***^	0.078^***^	-0.073^***^
	(0.014)	(0.010)	(0.011)	(0.012)	(0.016)
Live weight squared	-0.002^***^	-0.002^***^	-0.002^***^	-0.001^**^	0.002^***^
	(<0.001)	(<0.001)	(<0.001)	(<0.001)	(<0.00)
Male sheep	0.013	0.033	-0.026	0.032	-0.010
	(0.036)	(0.028)	(0.031)	(0.041)	(0.039)
B/n half and one year	-0.013	0.023	0.007	-0.187^***^	-0.222^***^
	(0.022)	(0.023)	(0.037)	(0.063)	(0.074)
B/n one and two years	0.007	0.020	0.049	0.137^***^	-0.176^**^
	(0.016)	(0.022)	(0.032)	(0.057)	(0.087)
B/n two and three years	0.014	-0.009	0.035	0.180^***^	0.211
	(0.022)	(0.026)	(0.032)	(0.067)	(0.154)
Above three years	-0.006	0.002	-0.006	0.152^***^	0.374^***^
	(0.028)	(0.026)	(0.031)	(0.061)	(0.130)
Very good	0.199^***^	0.194^***^	0.220^***^	0.161^***^	0.188^***^
	(0.042)	(0.034)	(0.041)	(0.051)	(0.051)
White	0.015	0.027	0.022	0.043	-0.030
	(0.027)	(0.022)	(0.029)	(0.033)	(0.044)
Brown	-0.011	-0.016	-0.027	-0.043^**^	-0.011
	(0.021)	(0.018)	(0.023)	(0.022)	(0.034)
Trader	0.031	0.006	-0.034	-0.060^**^	-0.066^**^
	(0.023)	(0.020)	(0.023)	(0.028)	(0.032)
Consumer	0.003	0.014	0.056^**^	0.034	0.189^***^
	(0.018)	(0.021)	(0.026)	(0.035)	(0.046)
**Month of purchase**					
October	0.059	0.017	0.009	0.007	0.024
	(0.054)	(0.041)	(0.050)	(0.051)	(0.067)
November	-0.010	-0.035	-0.079	0.010	0.020
	(0.065)	(0.055)	(0.049)	(0.066)	(0.091)
December	0.023	-0.004	-0.046	-0.156^***^	-0.072
	(0.042)	(0.038)	(0.054)	(0.051)	(0.064)
January	-0.101	-0.126^***^	-0.074^*^	-0.044	0.037
	(0.052)	(0.041)	(0.043)	(0.055)	(0.062)
February	-0.006	-0.021	-0.101	-0.144^***^	0.081
	(0.054)	(0.058)	(0.080)	(0.064)	(0.082)
March	0.048	0.077^**^	0.046	-0.021	-0.013
	(0.038)	(0.033)	(0.038)	(0.055)	(0.058)
April	0.057^**^	0.042	0.041	0.112^***^	0.035
	(0.025)	(0.028)	(0.034)	(0.043)	(0.057)
May	-0.001	0.020	0.036	-0.025	-0.036
	(0.040)	(0.030)	(0.034)	(0.040)	(0.055)
June	-0.070	0.055^*^	0.050	0.030	0.033
	(0.053)	(0.032)	(0.044)	(0.062)	(0.069)
July	-0.032	-0.044	0.015	0.090	0.005
	(0.056)	(0.046)	(0.043)	(0.059)	(0.061)
August	0.043	0.039	0.060^*^	0.088^*^	-0.044
	(0.033)	(0.037)	(0.034)	(0.051)	(0.056)
Market fixed effects	-0.078	-0.113^***^	-0.098^**^	-0.055	-0.121
	(0.047)	(0.037)	(0.046)	(0.056)	(0.066)
Constant	3.61^***^	3.39^***^	3.65^***^	4.09^***^	6.08^***^
	(0.176)	(0.123)	(0.138)	(0.163)	(0.192)
R-squared	0.161	0.376	0.431	0.574	0.404
Observations	1153	1153	1153	1153	1153

Notes: In all estimations, we included market fixed effects to control for unobserved market specific attributes. The 50^th^ quantile estimate is similar to the WLS point estimate (Table 2), except for a few variables. For example, the effects of the age of sheep are much stronger at the higher quantiles, with the WLS estimates far from the 50^th^ quantile estimate. Figures in the parentheses are bootstrapped standard errors (200 resampling); and *, **, and *** denote statistical significance at the level of 5%, 1%, and 0.1% statistical error, respectively.

The marginal effects of body condition are positive and statistically significant across all price quantiles. While statistically significant, the magnitude of the marginal effects of sheep body condition varies from quantile to another. The marginal effects are relatively higher in the lower quantiles implying that improving the body condition from *good* to *very good* will have a more pronounced distributional effect that favors sheep that fetch lower prices. Producers and consumers are the buyers who are purchasing the heaviest animals, and they are the ones paying the smallest price for the sheep (Table 2). This could explain the penalty for heavier weights at the upper end of the price distribution as producers and consumers show less interest when weight increases beyond a certain level [29].

The estimated unconditional partial effects also show that age categories affect the price distribution above the median. Sheep aged less than two years (cf. less than six months) are lower at the upper end of the price distribution. Hence, targeting the group that is willing to pay high for sheep with young sheep will not be useful to sheep sellers. On the other hand, buyers are willing to pay more for sheep aged above three years (cf. less than six months). This again reveals important market segments that could be targeted with sheep of different age groups.

Another interesting result beyond that of sheep traits is the heterogeneous effect on the types of sheep buyers. There is a clear preference heterogeneity among the different buyers at the upper end of the price distribution. Compared to producers, consumers are particularly willing to pay a higher price in the upper quantiles, whereas traders are significantly less interested (cf. producers) in paying a higher price in the upper quantiles. A possible explanation is that traders may possess greater knowledge than consumers, which is considered the norm in most markets in developing countries [12, 30]. Given that the markets being analyzed are characterized by information asymmetry and short market durations, traders would have an upper hand in bargaining power over other actors, ultimately allowing them to get price discounts. We also find that the coefficients of the selling seasons tend to vary in some quantiles. Sheep transactions from November to February (cf. September) command price discounts at different quantile levels, while price premium is observed when the transactions are made in March, April and August. The results provide evidence of the significance of enhancing farmers’ capacity to respond to seasonal changes in the sheep market. For example, availability of synchronization methods and feeding technologies to farmers help them to exercise planned lambing and marketing [31].

## 5. Conclusions

The lack of information on the heterogeneity of the effects of animal traits on observed prices is an important gap in the development of policies for improving markets and marketing in Ethiopia. In this study, we hypothesized that the traits of sheep affect the observed price differently depending on the different values of the traits. We used the FGLS and (un)conditional quantile regression models to examine the role of buyers and sheep attributes in determining the observed prices of sheep. Our study overall has two important findings. The first is that animal traits, buyer attributes, and seasonality of sheep marketing result in sheep price differences. Another important finding is the heterogeneous effects of animal traits on the observed price of sheep.

The empirical results confirm that livestock market modelling deviates from the classical consumer theory, which posits that consumer utility is derived only from the characteristics of the products [11]. In addition to sheep traits, the price of sheep is a function of the time of transactions, implying the importance of access to price information for farmers to respond to seasonal changes in livestock markets. Buyers are willing to pay a higher price per kilogram for sheep that are not fatty (cf. fatty ones) over all price ranges. They also pay the highest price per kilogram for sheep marketed at the age of a year while paying the lowest per kilogram for sheep marketed at the age of more than three years. This finding highlights the need for technologies that improve the dressing percentage of sheep at a young age so that sheep keepers receive better prices.

The findings also suggest applying improved husbandry practices and breeding schemes, as well as investing in the livestock market development. For example, availing appropriate fattening technologies to farmers targeting holiday markets is an ideal investment that will certainly improve their income. Similarly, the development of market infrastructure that significantly reduces the adverse effects of asymmetric information will potentially increase farmers’ share of market benefits.

Finally, we highlight a couple of limitations of our study. First, we used data only from primary rural markets and hence we could not analyze the heterogeneous effect of different values of animal traits at higher-level markets. Second, we did not account for the seasonality of sheep marketing, which could partially explain the observed price heterogeneity.

## Supporting information

S1 Appendix (PDF)
