# Supplementary material for: Willingness to pay for sheep traits and their heterogeneous effects on prices: Evidence from primary livestock markets in Ethiopia
Source: PLoS One. 2024 Sep 19;19(9):e0308651. doi: 10.1371/journal.pone.0308651 (PMC11412635; doi:10.1371/journal.pone.0308651)
Supplement: S1 Appendix — (PDF) [file pone.0308651.s001.pdf]

Appendix

Table A1. Number of sheep sold to different market actors

Variable	Producer	Trader	Consumer
Very thin sheep (< =10 kg)	55	51	9
Thin sheep (>10-20 kg)	203	368	121
Moderate sheep (20-30 kg)	70	70	36
Fat sheep (>30 kg)	57	28	35

Table A2. Test for heteroscedasticity

Variable	**Chi2**	***p*-value**
Live weight	0.01	0.909
Live weight squared	0.01	0.904
Sex of the animal	0.04	0.839
B/n half and one year	3.74	0.053
B/n one and two years	2.24	0.135
B/n two and three years	5.57	0.018
Above three years	8.01	0.005
Body condition	0.01	0.933
White	1.28	0.258
Brown	0.21	0.646
Traders	0.06	0.810
Consumers	23.53	0.000
October	1.46	0.226
November	0.82	0.366
December	0.01	0.921
January	1.08	0.299
February	0.02	0.884
March	3.64	0.057
April	10.05	0.002
May	3.53	0.060
June	0.34	0.558
July	0.74	0.391
August	0.31	0.575
Market place	0.68	0.411
Simultaneous	96.52	0.000

Notes: The Breusch-Pagan test is used to check the distribution of error terms. The *p*-value of the model, in general, reveals that the constant variance hypothesis is rejected, indicating that the distribution of the error terms is heteroscedastic.

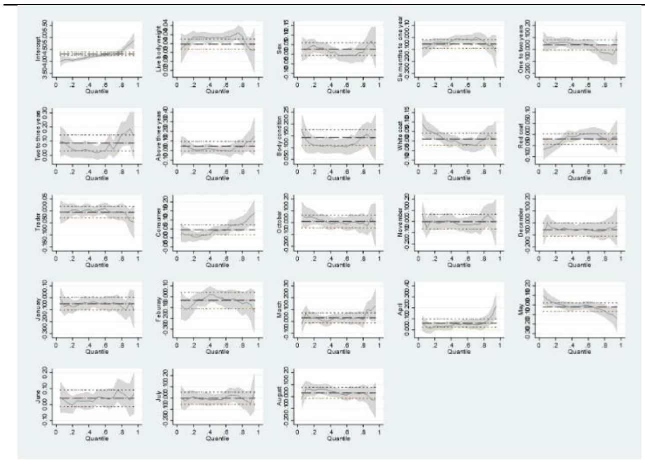

Figure A1. Quantile distributions of animal traits and other variables

Notes: the quantile distributions illustrate how the marginal effects of each animal trait vary across quantiles.
